# Limbic Encephalitis as a Presenting Complication for Small Cell Lung Cancer

**DOI:** 10.7759/cureus.9623

**Published:** 2020-08-09

**Authors:** Ahmad Abu-Hashyeh, Abdulrahman Katabi, Fuad Zeid

**Affiliations:** 1 Internal Medicine, Joan C. Edwards School of Medicine, Marshall University, Huntington, USA; 2 Pulmonary Medicine, Joan C. Edwards School of Medicine, Marshall University, Huntington, USA

**Keywords:** paraneoplastic neurological syndromes, small-cell lung carcinoma, status epilepticus, limbic encephalitis, autoimmune encephalitis

## Abstract

Limbic encephalitis (LE) is a rare neurological paraneoplastic complication that occurs secondary to malignant tumors. It is commonly presented as refractory seizures that are resistant to most anti-epileptics. We are presenting a unique case of small cell lung cancer complicated with LE. The challenging part of our case is that the patient had a history of seizure disorder in the past, and she was treated initially as an anti-epileptic treatment failure. A 68-year-old patient with a history of epilepsy was admitted to the ICU with resistant status epilepticus (SE), and respiratory failure secondary to pneumonia. Further workup revealed that the patient has small cell lung carcinoma. An extensive workup done to investigate resistant seizures revealed that she had a rare type of paraneoplastic autoantibodies (Anti-Hu) in the cerebrospinal fluid, which supported the diagnosis of the paraneoplastic autoimmune LE. High dose steroids helped to decrease the seizures episodes, but the family decided to proceed with palliative measures only at the end. Diagnosing LE requires ruling out other common causes of SE. Treatment options include treating underlying cancer as well as means of immunosuppression or antibody removal by tacrolimus and cyclophosphamide and even intravenous immunoglobulin (IVIG) or plasma exchange. It is important to consider LE in the differential diagnosis when managing patients with resistant SE in the ICU, even if the brain imaging and cerebrospinal fluid (CSF) analysis were within normal limits.

## Introduction

Paraneoplastic limbic encephalitis (LE) is an autoimmune-mediated disease that happens in association with different cancers. It is a rare complication that occurs in 1 per 10,000 patients [1]. Common cancers include lung cancer (50%), testicular cancer (20%), and breast cancer (8%) [2]. It is challenging to manage these cases, especially when the underlying cancer remains occult at the time of presentation. The main signs and symptoms of LE are loss of short-term memory, confusion, seizures, or psychiatric symptoms. 

## Case presentation

**Figure 1:**
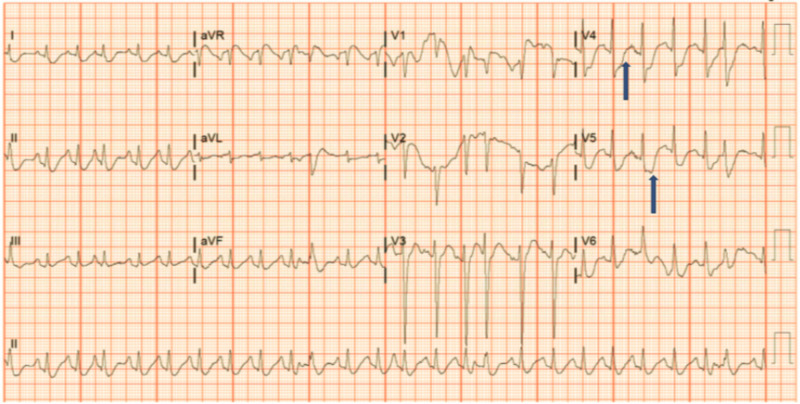
Initial EKG showing non-specific ST changes. EKG, electrocardiogram

**Figure 2:**
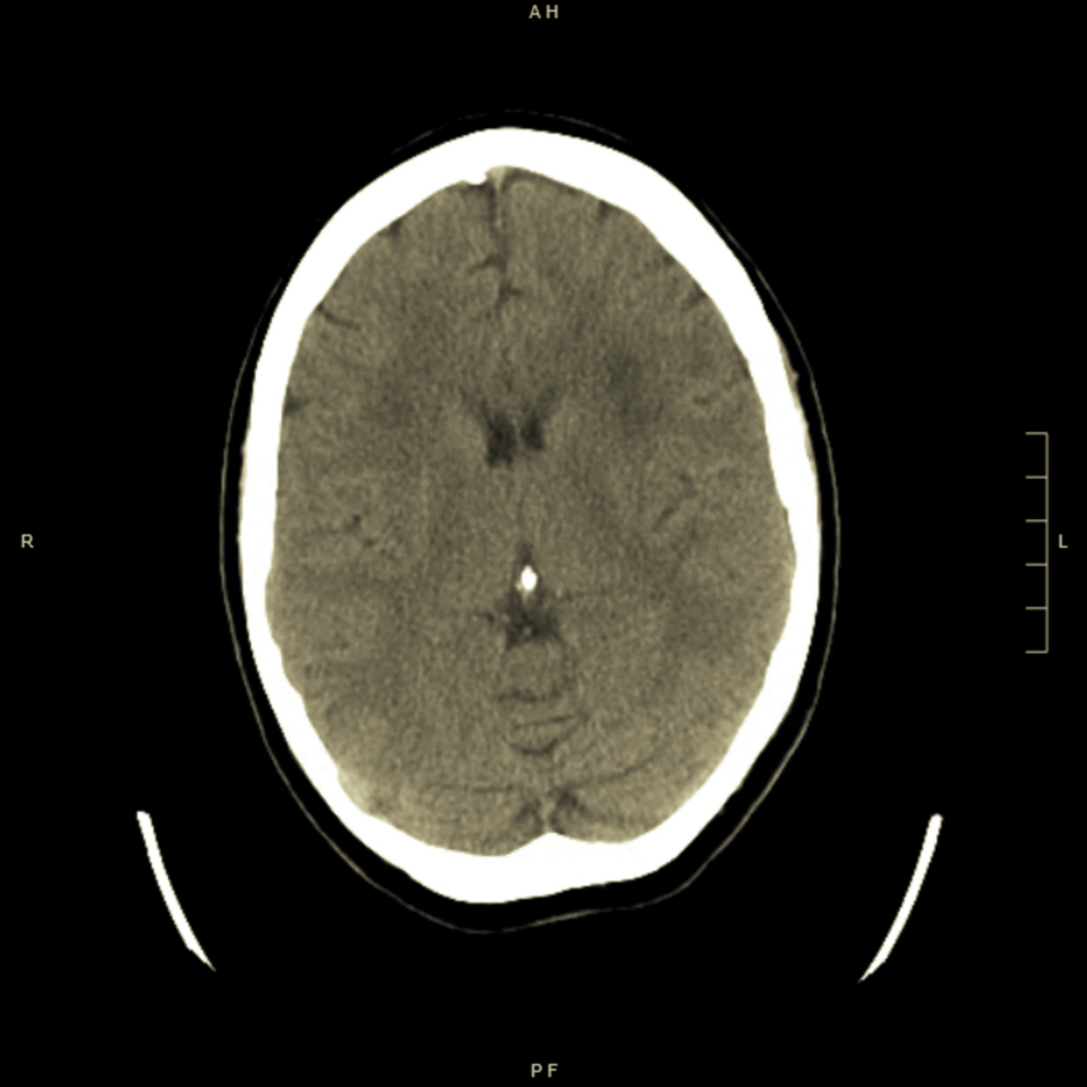
Head CT showing chronic ischemic changes.

**Figure 3:**
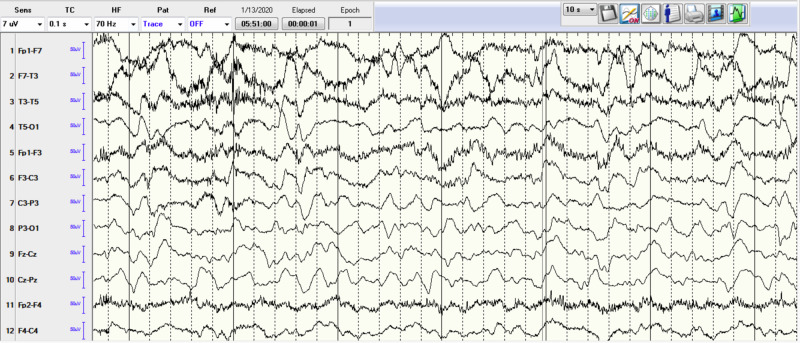
EEG showing rhythmic spikes. EEG, electroencephalography

**Figure 4:**
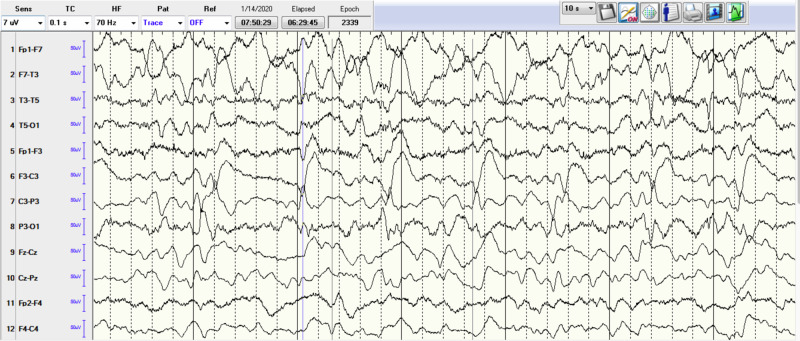
EEG showing rhythmic spikes. EEG, electroencephalography

A 68-year-old female patient with past medical history important for seizure disorder secondary to traumatic brain injury in the past, chronic obstructive pulmonary disease (COPD), cerebral aneurysm, type-2 diabetes mellitus, and hypertension, presented to the ER for acute respiratory distress and several days’ history of progressive altered mental status, repetitive epileptic movements, and recurrent falls without significant head injuries. She was not on any seizure-predisposing medications at home. The patient was intubated in the ER emergently for progressive severe hypoxic respiratory failure and for controlling status epilepticus (SE). Initial arterial blood gases on room air were pH 7.30, pCO2 56, and pO2 89. Other labs showed leukocytosis white blood cell (WBCs) 18.6 k/cmm with neutrophils predominance, elevated lactic acid at 4.3 mmol/L (normal range 0.7-2.1), hypokalemia at 2.5 mEq/L, and hypomagnesemia at 1 mg/dL. Troponin-I trended from 516 to 1615 pg/mL. Initial electrocardiogram (EKG) showed diffuse ST-segment depression (Figure 1). Chest X-ray showed right lower lobe infiltrate. Head CT scan without contrast showed chronic small vessel ischemic disease, without acute pathological changes (Figure 2). Brain MRI showed chronic small vessel cerebrovascular disease as well. Electroencephalography (EEG) demonstrated left rhythmic hemispheric spikes without evolving into seizures (Figures 3-4). Cardiac ECHO showed basal inferior hypokinesis with normal ejection fraction. She was admitted to the ICU for sepsis and acute respiratory failure secondary to pneumonia, SE, non-ST elevation myocardial infarction (NSTEMI), and multiple electrolyte abnormalities. Initial management included IV fluids, broad-spectrum antibiotics, ipratropium bromide/albuterol nebulizer, aspirin, atorvastatin, metoprolol, heparin drip, and levetiracetam. Electrolytes replaced as well. Sodium valproate was added later on due to repeated epileptic movements. 

The respiratory status initially improved, and the patient failed extubation trial and she required reintubation for severe stridor. Video EEG showed a picture of diffuse cerebral dysfunction with epileptogenicity in the temporal regions and interictal activity in the left hemisphere. The patient was difficult to wean from the ventilator after the second intubation. CT chest with IV contrast was done to investigate extubation failure and it ruled out pulmonary embolism but it showed right paratracheal lymph node measuring 3.6 cm, subcarinal lymph node measuring 5.9 cm, and left hilar lymph node measuring 2.8 cm highly suspicious for malignancy. Endobronchial ultrasound (EBUS) was performed to biopsy the lymph nodes, and it showed no endobronchial abnormalities. The lymph nodes biopsy pathology result was positive for small cell carcinoma with positive for CD56 and synaptophysin, and negative for CD20. Brain MRI showed no acute abnormalities. Lumbar puncture was performed, cerebrospinal fluid (CSF) studies showed: opening pressure 10 cm H2O, clear CSF, Protein 44, Glucose 110, negative meningitis panel, and negative for malignant cells. Paraneoplastic evaluation of the CSF identified anti-neuronal nuclear antibody type-1 (Anti-Hu) 1:512 (reference <1:2), which supported the diagnosis of paraneoplastic autoimmune neurological disorder. Bone scan and abdomen and pelvic CT with IV contrast both showed no metastases. IV methylprednisolone 1000 mg daily was started and the seizures were controlled. At this point, the family decided to proceed with palliative measures only without active cancer treatment. Percutaneous endotracheal tracheostomy (PEG) tube was inserted due to multiple extubation trial failures. Also, a PEG tube was inserted before discharging the patient to a long-term acute care facility.

## Discussion

Limbic encephalitis is a paraneoplastic disorder that is described in many types of cancer; common culprits include lung cancer, testicular cancer, ovarian cancer, and breast cancer (usually within a four-year period) but it has also been described in many others. Although the diagnosis can rarely precede the appearance of cancer in a small group of patients, and while presentations tend to vary from one case to the other, it is usually a disease that progresses in a subacute fashion and has symptoms ranging from seizures to neuropsychiatric complaints of depression and anxiety. Diagnosing paraneoplastic LE requires ruling out other common causes of this disease like autoimmune LE [3].

Multiple antibodies can be identified in the CDF and are sometimes related to the prognosis of the disease. Common antibodies include (anti-NMDAR, anti-Hu, anti-Yo among many others). Lack of these antibodies does not rule out the disease given that a minority of cases (10%-20%) can have a seronegative disease, which appears to carry a slightly worse prognosis.

MRI can be helpful in diagnosis by showing amygdala-hippocampal signals especially in fluid-attenuated inversion recovery (FLAIR), and some temporal atrophy may be seen later in the course of the disease. These changes can be found in around 50%-60% of the cases and sometimes MRIs need to be repeated as they may be normal in the early stages of the disease [4].

Of note, the seizures accompanying LE can be refractory to a wide variety of anti-epileptic drugs as in our case due to the origin of the seizures being in the medio-temporal lobe, which was the trigger for the extensive evaluation that was pursued in our case after having resistant severe seizures and an EEG concerning for temporal lobe seizures [5].

Paraneoplastic autoimmune encephalitis can be treated with an approach that focuses on treating underlying cancer as well as means of immunosuppression or antibody removal. Medications include steroids tacrolimus and cyclophosphamide and even intravenous immunoglobulin (IVIG) or plasma exchange in some cases. The choice of therapy may be influenced by the location of the antibody (intra-cellular vs extra-cellular) [6].

## Conclusions

Paraneoplastic LE is a rare condition that presents with common symptoms or signs like seizures or altered mental status. Although LE is a rare paraneoplastic complication, it is important to consider it in the differential diagnosis list when managing patients with resistant SE in the ICU even if the head imaging and CSF analysis were within normal limits. 
